# Sublethal Damage Caused by Cold Plasma on *Bacillus cereus* Cells: Impact on Cell Viability and Biofilm-Forming Capacity

**DOI:** 10.3390/foods13203251

**Published:** 2024-10-13

**Authors:** Laura Eced-Rodríguez, Michael Beyrer, Dolores Rodrigo, Alejandro Rivas, Consuelo Esteve, Maria Consuelo Pina-Pérez

**Affiliations:** 1 Departmento de Microbiologia y Ecologia, Facultad de Ciencias Biológicas, Universitat de València, 46010 Valencia, Spain; lauegu@alumni.uv.es (L.E.-R.); maria.esteve@uv.es (C.E.); 2 Department of Natural Products, Institute of Life Technologies, HES.SO Valais-Wallis, 1950 Sion, Switzerland; michael.beyrer@hevs.ch; 3 Departamento de Conservación y Calidad de los Alimentos, IATA-CSIC, 46980 Paterna, Spain; 4 Departamento Tecnología de Alimentos, Instituto Universitario de Ingeniería de Alimentos-FoodUPV, 46022 Valencia, Spain; alriso@upvnet.upv.es

**Keywords:** sublethal damage, cold plasma, *Bacillus cereus*, viability, biofilm, food safety, powder products

## Abstract

The *Bacillus cereus* group represents a serious risk in powdered and amylaceous foodstuffs. Cold plasma (the fourth state of matter) is emerging as an alternative effective nonthermal technology for pasteurizing a wide range of matrices in solid, liquid, and powder form. The present study aims to evaluate the mechanisms involved in *Bacillus cereus* inactivation via cold plasma, focusing on (i) the technology’s ability to generate damage in cells (at the morphological and molecular levels) and (ii) studying the effectiveness of cold plasma in biofilm mitigation through the direct effect and inhibition of the biofilm-forming capacity of sublethally damaged cells post-treatment. Dielectric barrier discharge cold plasma (DBD-CP) technology was used to inactivate *B. cereus*, *B. thuringiensis*, and *B. mycoides* under plasma power settings of 100, 200, and 300 W and treatment times ranging from 1 to 10 min. Inactivation levels were achieved in 2–7 log_10_ cycles under the studied conditions. Percentages of sublethally damaged cells were observed in a range of 45–98%, specifically at treatment times below 7 min. The sublethally damaged cells showed poration, erosion, and loss of integrity at the superficial level. At the molecular level, proteins and DNA leakage were also observed for *B. cereus* but were minimal for *B. mycoides*. Biofilms formed by *B. cereus* were progressively disintegrated under the DBD-CP treatment. The greater the CP treatment intensity, the greater the tearing of the bacteria’s biofilm network. Additionally, cells sublethally damaged by DBD-CP were evaluated in terms of their biofilm-forming capacity. Significant losses in the damaged cells’ biofilm network density and aggregation capacity were observed when *B. cereus* was recovered after inactivation at 300 W for 7.5 min, compared with the untreated cells. These results provide new insights into the future of tailored DBD-CP design conditions for both the inactivation and biofilm reduction capacity of *B. cereus* sensu lato species, demonstrating the effectiveness of cold plasma and the risks associated with sublethal damage generation.

## 1. Introduction

At present, market trends have shifted to highly convenient and nutritious products targeted at specific consumers (e.g., the sports sector, the elderly, infants, allergenic people). In this context, powdered products are gaining attention due to their ease of handling and transportation, good microbiological and nutritional quality that can be maintained over years, and their ability to be preserved at room temperature. Nutraceutical products, including algae-derived bioactives, vegetable proteins and fibers, dairy concentrates, and whole cereals and brans, are being delivered in powdered form to satisfy food, pharmaceutical, and cosmetic industrial requirements [1]. However, these matrices are not sterile, and most of the bioactive contents are thermolabile (i.e., not suitable for processing at high temperatures).

*Bacillus cereus* threatens the safety of dried and powdered products such as herbs, species, and powdered milk (especially powdered infant milk formula) and is present in cereal flour (rice, wheat, and soya). *Bacillus cereus* belongs to the *B. cereus* group of bacteria, also known as *B. cereus* sensu lato (s.l.), consisting of eight closely related species, namely *B. anthracis*, *B. cereus* sensu stricto (s.s.), *B. cytotoxicus*, *B. mycoides*, *B. pseudomycoides*, *B. thuringiensis*, *B. toyonensis*, and *B. weihenstephanensis* [2].

*Bacillus cereus* is a spore-forming bacterium that can persist in industrial environments and facilities. Additionally, *B. cereus* can adapt to other conditions when forming biofilms, which is of utmost importance regarding its persistence in food industry equipment, such as processing pipelines. Biofilms protect spores and vegetative cells against chemical and physical treatments. Biofilms are assemblages of microbial cells attached to each other and industrial equipment and, in most cases, they are the cause of contamination in final products. In fact, it is believed that most pathogens exist on biotic and abiotic surfaces in the biofilm matrix instead of the planktonic form [3]. Specifically, in the food industry, modern food-processing lines are a suitable environment for biofilms to form on food contact surfaces, primarily because of the complexity of manufacturing plants, long production periods, mass product generation, and large biofilm growth areas [3]. In existing investigations, the most frequent approaches for biofilm prevention are based on (i) blocking bacterial adhesion, (ii) killing persistent cells, (iii) interfering with bacterial communication, (iv) inhibiting bacterial cooperation, (v) degrading the exopolysaccharide (EPS) matrix, and (vi) stimulating dispersal [4]. Physical and chemical treatments are mainly applied to industrial surfaces and medical–surgical instruments/environments to remove biofilms and persistent cells. In these treatments, cold plasma (the fourth state of matter, including charged particles, ions, atoms, active species, free radicals, molecules, and UV radiation) is emerging as a sustainable and effective physicochemical technology, which can inactivate bacteria in both vegetative and sporulated form, as well as impacting biofilm formation. Cold plasma accomplishes this in the following ways:

1. Surface functionalization and coating (avoiding subsequent bacterial adhesion) [5].
2. Directly treating biofilms and their detaching procedures through sequential effects: First, it destroys the biofilm matrix through active species—mainly reactive oxygen species (ROS) and reactive nitrogen species (RNS)—breaking down bonds in hydrocarbon compounds, which collapses and destroys the EPS biofilm matrix and the protein structure and enzymatic activity of microorganisms in the biofilm’s architecture. Second, once the biofilm structure has been seriously affected, the microorganisms embedded in it are moved to a bulk solution, with some adopting a planktonic form. In parallel, active species, charge ions, and UV radiation affect the membrane integrity of the microorganisms, interfering with their cellular metabolic pathways, promoting intracellular material leakage, and finally killing the microorganisms in the biofilm. At the same time, the biofilm matrix separates from the biotic and abiotic surfaces, eliminating it on the solid substratum [6,7].

The effects of cold plasma on biofilm formation—destruction, density reduction, metabolic activity in bacteria immersed in biofilm, and even *quorum-sensing* inducers—have been studied in many bacteria, including *P. aeruginosa* [8], *Staphylococcus aureus* [9], and *Bacillus subtilis* [10]. *Listeria monocytogenes*, *Pseudomonas fluorescens* [11], and *Escherichia coli* O157:H7 [12] are among the best-studied biofilm-forming bacterial pathogens in foods that have been effectively inactivated by CP.

The present study assesses the ability of DBD-CP to sublethally damage *Bacillus cereus* s.l. group cells, focusing on quantifying (i.e., percentages of sublethal damage) and assessing any damage generated by each treatment at the structural and molecular levels (based on a combination of power and treatment time). Additionally, test monoculture biofilms are developed, and three aspects are evaluated: (i) the potential of DBD-CP to affect the biofilm structure, (ii) the viability of biofilm-immersed cells after treatment, and (iii) the capacity of sublethally damaged cells to form biofilms.

## 2. Materials and Methods

### 2.1. Bacterial Strains and Growth Conditions

For the present study, three species were selected within the *B. cereus* s.l. group: *B. cereus*, *B. thuringiensis*, and *B. mycoides*. Bacterial strains were provided by the Spanish Culture Type Collection (CECT), including *B. cereus* CECT 4387; *B. cereus* CECT 7259; *B. thuringiensis* CECT 197 T; and *B. mycoides* CECT 4128.

Lyophilized cultures were revived according to the CECT instructions. Briefly, lyophilized cells were recovered in 10 mL of brain heart infusion broth (BHIB) (Scharlab S.A., Barcelona, Spain) and maintained under agitation at 30 °C for 20 min to favor cell reactivation. Afterward, this 10 mL was transferred to BHIB (200 mL) in a sterile flask and kept under optimum incubation conditions for 48 h. Cells were harvested via centrifugation (8000 rpm, 20 °C, 10 min) and washed 3 times with sterile BHIB. Then, pellets were resuspended on sterile BHIB and dispensed in cryovials, including 1 mL of the pure culture suspension and 1 mL of glycerol in BHIB (10% *v*/*v*) (stock final concentration: 1010 CFU/mL).

### 2.2. Cold Plasma Equipment Used

A dielectric barrier discharge–low-pressure plasma device was used (Electronic Diener Plasma Surface Technology PCCE, model Pico-AR-200-PCCE7). Plasma is generated through two circular plate electrodes operating at a frequency of 13.56 MHz and a power range from 0 to 300 W.

Suspensions of *B. cereus*, *B. thuringiensis*, and *B. mycoides* vegetative cells (10^8^ CFU/mL) were prepared and distributed on sterile glass slides (1 mL of culture per slide, 76 × 20 mm) and subjected to the plasma treatment. Slides were exposed to forced cabinet airflow to dry the cell suspensions. Cells attached to the glass slides were immediately processed using CP under different conditions: 100, 200, and 300 W (1–7.5 min). The experiments were performed with pure oxygen (O_2_) as the working gas, ignited at room temperature (23 ± 2 °C).

### 2.3. Microbiological Enumeration

The surfaces of the inoculated glass slides (treated samples and controls) were scraped with a sterile spatula, and cells were recovered on sterile Petri dishes by adding phosphate-buffered solution (PBS, Merck KGaA, Darmstadt, Germany). After vigorous agitation, glass slides were separated, and aliquots were taken from suspensions (including control cells or treated cells) to carry out serial decimal dilutions to count bacteria in the BHIA plates. Plates were incubated at 37 °C for 48 h before counting bacteria-grown colonies (CFU/mL).

Bacterial inactivation via CP is expressed as log_10_ (Nf/N_0_): where Nf is the bacterial load after treatment (CFU/mL) and N_0_ is the initial bacterial count (CFU/mL).

### 2.4. Sublethal Damage Evaluation

#### 2.4.1. Sublethal Damage Quantification

Just after the CP treatment, 0.1 mL aliquots of each decimal dilution (Section 2.3) were plated on selective and nonselective agar. BHIA was used as nonselective agar, enabling the growth of both viable and damaged cells. Meanwhile, BHIA supplemented with 3% NaCl (*w*/*v*) was used as a selective medium, on which only the viable non-damaged cells were allowed to grow [12]. The differences between cell counts on the nonselective and selective media were used to estimate sublethally damaged cells. The percentage of sublethal damage was estimated with the following equation (Equation (1)):

Sublethally damaged cells (%) = ((cell number in nonselective media − cell number in selective media))/(cell number in nonselective media) × 100(1)

In parallel, CP-treated and control replicates were transferred to BHIB to follow up the growth behavior after processing at 30 °C for 24 h. Aliquots were plated in BHIA + 3% NaCl and BHIA to differentiate viable and damaged cell evolution during storage.

#### 2.4.2. Sublethal Morphological Damage Assessed by Scanning Electron Microscopy (SEM)

Treated cells exposed to plasma (and controls) were directly analyzed with SEM. Micro-glass slides were used (ϕ = 8 mm). Aliquots (20 µL) of bacterial cultures were scattered on the slides and dried. Immediately, cells were exposed to plasma and subsequently fixed for microscopic observation. Following fixation for 2 h in ice-cold 2.5% glutaraldehyde, samples were treated with increasing concentrations of ethanol (30%, 50%, 70%, 80%, 95%, and 99.5%) and dehydrated using 33%, 50%, 66%, and 100% (*v*/*v*) hexamethyldisilazane (Sigma Aldrich, Arklow, Ireland). Samples were sputter-coated with gold particles using an Emitech K575X Sputter Coating Unit, resulting in a 10 nm coating after 30 s. The samples were examined visually using a Hitachi S-4800 SEM device at 1 kV (located at the University of Valencia—SCSIE (Servicio Central de Soporte a la Investigación Experimental)).

#### 2.4.3. Cell Membrane Integrity and Intracellular Content Leakage

To detect bacterial cell membrane integrity loss and the release of intracellular proteins and nucleic acids after the DBD-CP treatment, DNA and protein concentrations were spectrophotometrically quantified by recording UV absorbances at 260 and 280 nm, respectively [13]. Surviving cells after each CP treatment (and control cells without treatment) were recovered in PBS and incubated at 37 °C for 1 h under agitation (200 rpm) and then centrifuged (12,000× *g*, 15 min). Supernatants were transferred to a quartz cell to determine the UV absorbances at 260 nm and 280 nm. All measurements were taken in triplicate with a UV-1800 spectrophotometer (HiPo MPP-96, Biosan, Riga, Latvia). Recordings were expressed as percentages of the extracellular UV-absorbing materials released by the cells.

### 2.5. Impact of CP on Biofilm-Forming Capacity

#### 2.5.1. Screening Biofilm-Forming Capacity in *Bacillus cereus* s.l. Group Cells

*B. cereus* 4387 CECT, *B. cereus* 7259 CECT, *B. thuringiensis* 193T, and *B. mycoides* 4128 CECT were tested for their biofilm-forming capacity [14]. Fresh bacterial cultures were prepared on sterile BHIB (10 3 CFU/mL) and incubated at 37 °C for 24 h. Afterward, a 1:100 dilution was carried out for each bacterial culture, and 200 µL aliquots were disposed of on individual 96-well polystyrene microtiter plates (in triplicate). Plates were incubated for 48 h at 37 °C to facilitate biofilm formation. After incubation, the supernatant in each well was removed. Phosphate–saline buffer (PBS, Merck KGaA, Darmstadt, Germany) was used to wash each well twice. Considering that biofilm was present at the bottom of each well, 200 µL of 95% ethanol was added to fix the cells to the bottom. Alcohol was removed, and cells were dried in air. Crystal violet staining agent (1% *v*/*v*) was added to each well and maintained contact with the biofilm for 30 min. Then, the staining agent was removed, and 3 washing steps were carried out with distilled water. An ethanol–acetone mixture [70:30] was added to each well to solubilize the staining agent retained by the biofilm. The absorbance was read at 630 nm with a HIPO MPP-96 Microplate Photometer (Biosan S.L., Riga, Latvia).

#### 2.5.2. SEM Visualization of Biofilms Formed by Sublethally Damaged Cells after DBD-CP

Surviving *B. cereus* cells after the DBD-CP application were recovered and incubated under optimum conditions (37 °C, 48 h) to promote biofilm formation on micro-glass slides (ϕ = 8 mm) immersed in BHIB on borosilicate Petri dishes (80 mm). DBD-CP treatment conditions were selected to guarantee sublethally damaged percentages of 70–98%. Control biofilms (from untreated cells) and biofilms formed by recovered populations exposed to different DBD-CP conditions (including sublethally damaged cells) were dried and fixed on ice-cold 2.5% glutaraldehyde. The biofilms were dehydrated using sequential immersion on 33%, 50%, 66%, and 100% (*v*/*v*) alcohol and sputter-coated with gold particles using an Emitech K575X Sputter Coating Unit, resulting in a 10 nm coating after 30 s. The samples were visually examined using an FEI Quanta 3D FEG Dual Beam SEM (FEI Ltd., Hillsboro, OR, USA) at 5 kV. Density, integrity, and cell agglomeration into biofilms were inspected in the SEM images.

#### 2.5.3. SEM Visualization of Biofilms Directly Damaged by DBD-CP

Biofilms were formed on micro-glass slides (ϕ = 8 mm) (6 per plate) immersed into BHIB Petri dishes. Control-grown and DBD-CP-treated biofilms on the slides were then dried and fixed on ice-cold 2.5% glutaraldehyde according to previous exposure. Biofilms were dehydrated using sequential immersion on 33%, 50%, 66%, and 100% (*v*/*v*) alcohol and sputter-coated with gold particles using an Emitech K575X Sputter Coating Unit, resulting in a 10 nm coating after 30 s. The samples were visually examined using a Hitachi S-4800 SEM device at 1 kV (located at the University of Valencia—SCSIE). Density, integrity, and cell agglomeration into biofilms were inspected in the SEM images.

#### 2.5.4. Viability of Cells Immersed in Biofilms

The metabolic activity of bacterial cells within the biofilms was determined at three time intervals, (i) just after treatment, (ii) 8 h after treatment, and (iii) 24 h after treatment, using the 2,3-bis (2-methoxy-4-nitro-5-sulfophenyl) [phenyl-amino)carbonyl]-2H tetrazolium hydroxide assay (XTT, 1 mg/mL, Sigma-Aldrich Co., Wicklow, Ireland) [11].

This colorimetric assay reduces yellow tetrazolium salt into an orange formazan dye by metabolically activating cells. The formazan dye is soluble in aqueous solutions and can be directly quantified using a scanning multiwell spectrophotometer (HiPo MPP-96, Biosan, S.L., Riga, Latvia). An increase in living cells increases the overall activity of mitochondrial dehydrogenases in the sample. This increase directly correlates to the amount of orange formazan formed, as monitored by the absorbance at 486 nm.

Briefly, microslides containing biofilms and negative controls were transferred to falcon tubes filled with BHIB (10 mL) and submerged in an ultrasound bath (Digitec 100H, 35 kHz, Biosan, S.L., Riga, Latvia) for 5 min to disrupt biofilms. Aliquots (100 µL) from each sample–replicate subjected to each CP treatment condition (and controls) were mixed with fresh XTT solution (100 µL) in individual wells of sterile microtiter plates (96 wells), and an initial 486 nm picture was taken (t = 0 h after treatment) on a microplate reader (HiPo MPP-96, Biosan, S.A.). Plates were incubated for 8 h at 37 °C in the dark under agitation. Afterward, the absorbance was measured at 486 nm. The metabolic activity of the surviving bacterial cells in the biofilms was finally measured 24 h after treatment. In all cases, it was calculated as a percentage in relation to the metabolic activity detected in the untreated control cells immersed in the biofilms.

#### Statistical Analysis

Statistical analysis was performed using Statgraphics Centurion XIX. For each Bacillus strain, an analysis of variance (ANOVA) was carried out to detect significant differences between CP treatments in terms of inactivation, sublethal damage impairment, and biofilm disruption capacity. Means were compared according to Fisher’s least significant difference (LSD) method at the 0.05 level.

## 3. Results and Discussion

### 3.1. Inactivation and Injured Cell Analysis after DBD-CP

**Figure 1:**
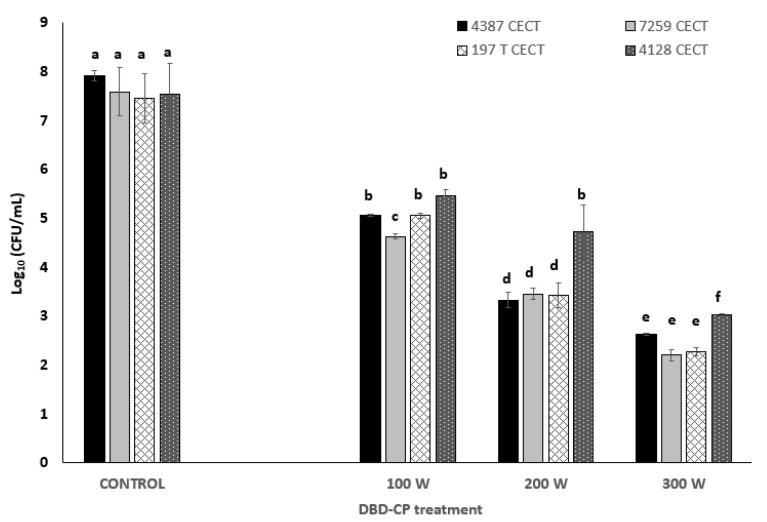
Inactivation levels caused by DBD cold plasma used against *Bacillus* sensu lato group (*B. cereus* 4387 and 7259 CECT, *Bacillus thuringiensis* 197 T CECT, and *B. mycoides* 4128 CECT) applying 100, 200, and 300 W for 7.5 min with pure O_2_ as the carrier gas. Different superscript letters (a–f) indicate significant differences (*p*-value < 0.05) between bars at each treatment condition.

Inactivation levels were obtained in 1 to 5 log_10_ cycles for *B. cereus* sensu lato group cells using DBD-CP under the studied treatment conditions. For each power level applied, the higher the treatment time (0–5 min), the higher the inactivation achieved (*p*-value ≤ 0.05). However, no significant differences (*p*-value > 0.05) in inactivation rates were detected between the 5 and 7.5 min treatments at any power level. Maximum inactivation levels close to 5 log_10_ cycles were achieved using DBD-CP at combinations of 300 W and 7.5 min using pure O_2_ as the carrier gas (Figure 1).

Inactivation levels close to three and two log_10_ cycles were generally obtained at 200 and 100 W, respectively, for all strains (using time treatments of 5–7.5 min). We can conclude that 5 min treatments at 100 W, 200 W, and 300 W power levels are the most efficient for achieving inactivation values of 2 to 5 log_10_ cycles using pure O_2_ as ignited gas for *B. cereus* 4387 CECT and 7259, *B. thuringiensis* 193T CECT, and *B. mycoides* 4128 CECT. *Bacillus mycoides* was the most resistant species to DBD-CP (*p*-value < 0.05), especially under high-intensity conditions (e.g., close to 2.5 log_10_ cycles: reduction at 200 W for 7.5 min).

Sublethal damage caused by DBD-CP was observed immediately after treatment. Additionally, the evolution of damaged cells versus total surviving cells after treatment was monitored over a complete 24 h under optimal growth conditions (inoculated in BHIB at 37 °C). The results confirmed different damage levels depending on the power CP intensity applied and the *Bacillus* species under consideration. Immediately after treatment, high damage levels were recorded in a range of 75–98% for 100, 200, and 300 W (5 min), considering all species under study. Damaged cells progressively decreased during storage (Table 1).

**Figure 2:**
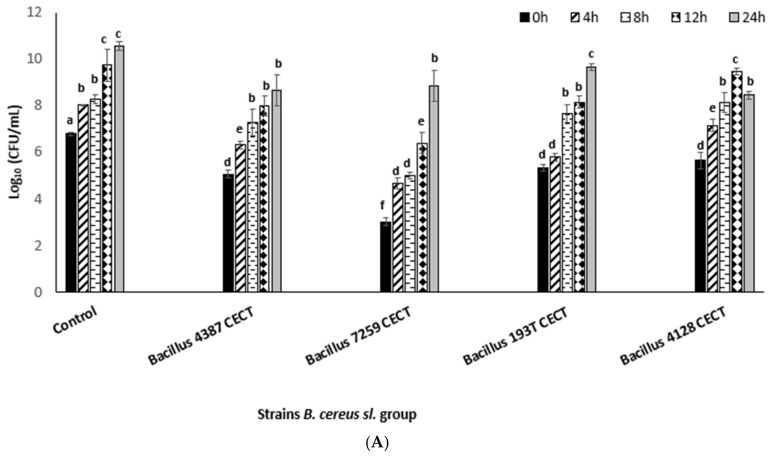

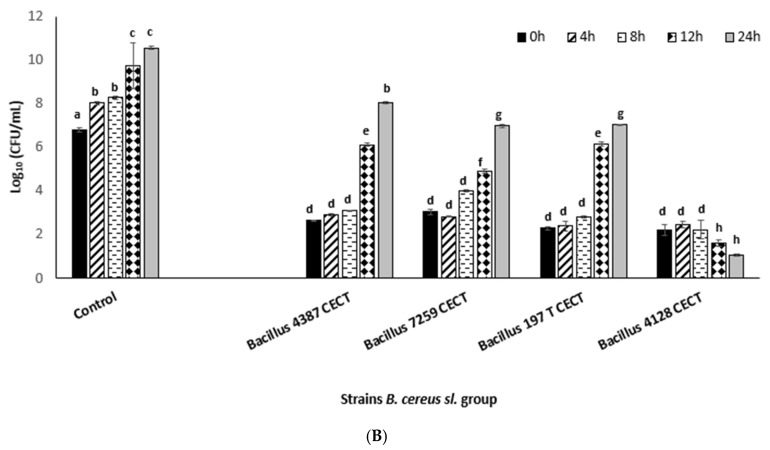
Evolution of recovered cells (viable + sublethally damaged cells) after DBD-CP treatment at (**A**) 100 W–5 min and (**B**) 300 W–5 min for *Bacillus cereus* (4387 CECT and 7259 CECT), *Bacillus mycoides* (4128 CECT), and *Bacillus thuringiensis* (197 T CECT), observed during incubation in brain heart infusion broth at 30 °C for 24 h. ^(a–h)^ Superscript letters above the bars indicate significant differences (*p*-value ≤ 0.05) between the decimal logarithms of the bacteria load levels for each strain in relation to the untreated cells.

However, the growth patterns of the surviving bacterial cells treated at 100 and 300 W differed significantly (*p*-value < 0.05). The *B. cereus* population progressively grew when the cells were exposed to the 100 W–5 min treatment (including the sublethally damaged cells) in a pattern very similar to the one observed for the untreated cells (controls) (Figure 2). The same occurred for cells exposed to the 200 W–5 min treatment (*p*-value > 0.05). Meanwhile, when the cells were exposed to the 300 W–5 min treatment, the progressive reduction in the percentage of sublethally damaged cells (Table 1) did not increase the global population (during 24 h incubation), probably because the progressive decay of sublethal damaged cells was irreversibly affected in comparison with the treated cells at 100 W (reversibly affected).

Although the mechanisms of microbial inactivation via CP have been well-defined as a combination of physical and biochemical damage, the complexity of this treatment and the microbial systems involved makes the sterilization process difficult to understand, or even to clearly describe. Over the last decade (2014–2024), many studies have focused on evaluating bacterial cell damage caused by cold plasma using different treatment conditions, bacterial species, and a wide variety of matrices [13,14,15,16]. Specifically, in food, it is crucial to better understand the cascade of events caused by CP inactivation that occurs during plasma treatment in order to accurately define possible associated risks to viable but nonculturable cells (VBNCs) or reversibly damaged cells generated in the process. To date, there is a lack of standardization for this process (many lab-scale devices used or being tested, parameters, and working gases, among other technological factors); tailor-made studies should be conducted for each technology and food product combination. Many studies focused on resuscitating cells after treatment have been carried out in the last few years, particularly with regard to elucidating VBNC cell-associated risks. According to Dolezalova and Lukes [17], *E. coli* enters a VBNC state after CP application (atmospheric pressure plasma jet, 1–5 kV, 1.5 MHz), retaining its virulent potential even 3 months after plasma treatment.

Liao et al. [18] suggested that DBD–atmospheric pressure CP-damaged cells (generated under input power values in a range of 40–60 W and treatment times of 0–45 s) are generated in significant proportions under low-intensity treatments, as the power intensity is the most influential factor on sublethal damage generation (the more intensive the treatment, the greater the inactivation). At the highest DBD power intensities (60 W–45 s), the lethal effect becomes very significant, as the proportion of sublethally damaged cells becomes negligible, for example, in *Staphylococcus aureus* treated indirectly under atmospheric pressure [19]. High levels of sublethal damage were achieved using DBD-CP in our study, independent of the plasma power intensity applied (range, 100–300 W).

Smet et al. [20] observed the recovery of *Salmonella Typhimurium* cells damaged by CP (99% He; 1% O_2_*;* 7.45 W) when they were inoculated in food simulation systems. The results showed that the recovery percentage after treatment was significantly higher when *S. Typhimurium* cells were incubated under optimum conditions (37 °C) after DBD-CP treatment. However, at 8 °C and 20 °C, the bacteria levels decreased during storage.

To the best of our knowledge, very few studies have examined recovered–damaged cells or their evolution in storage just after cold plasma treatments [13,14,15,16,20,21,22,23,24,25]. Our results show that damaged cells can completely recover their viability, increasing the global surviving population, under treatments with low power values (100–200 W). At the highest power intensities, some of the damaged cells seemed to die, with the surviving population’s growth pattern clearly different from the control’s growth pattern (untreated cells).

**Figure 3:**
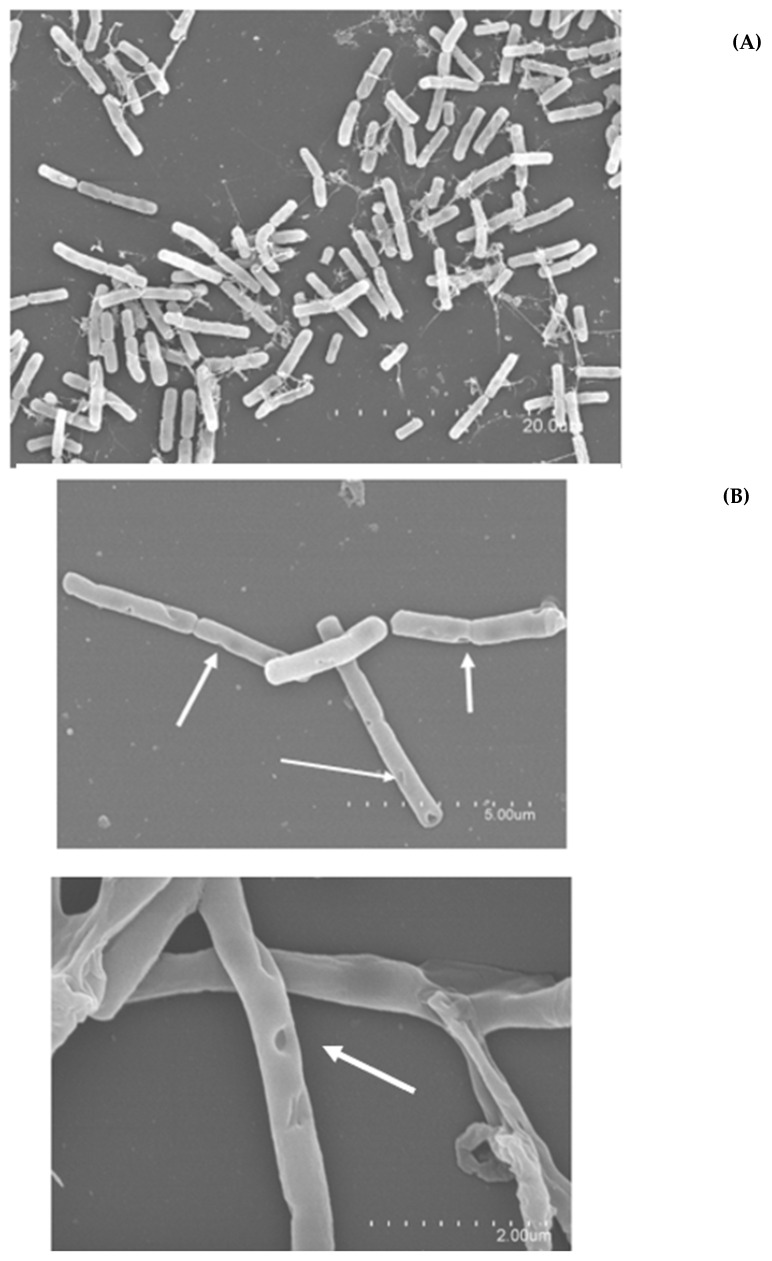

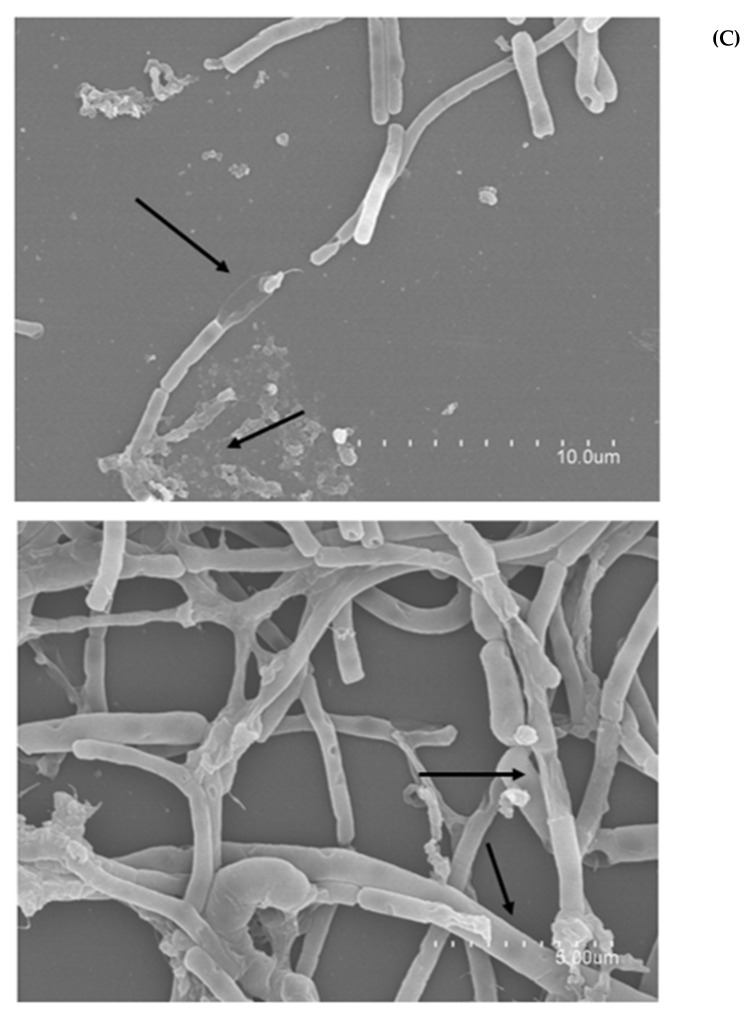
SEM images of control and treated *Bacillus cereus* (4387 CECT and 7259 CECT) cells subjected to 100, 200, and 300 W (5 min) DBD-CP treatments. (**A**) Controls. (**B**) White arrows indicate structural damage events (poration, erosion, excavation derived from reactive species, and charged particles on the bacterial surface) (100–200 W–5 min). (**C**) Black arrows indicate other events (elongation, flattening, collapse, and leakage) under the most intensive conditions applied (300 W–5 min).

To confirm the extent and quality of damage caused by DBD-CP, the resulting morphological damage and bacterial cell destruction were studied using SEM (Figure 3). The results regarding the formation of peak holes caused by CP, debris, and other cellular modifications are shown in Figure 3. With the formation of pores, cavities, and excavated holes, damage to the superficial cellular topography occurred under low-intensity treatments (100–200 W for 5 min) and appeared in a smaller proportion when a 5 min 300 W treatment was applied. Superficial and cell membrane damage can be considered the starting point in viability loss and microbial inactivation. This structural damage can be caused by the combined action of electrostatic forces; the accumulation of charged particles on the outer side of the membrane, which favors the erosion process; and the oxidative stress the cell is subjected to when exposed to ROS bombarding the cell surface [26]. According to Zhang et al. [26], the main mechanism affecting bacterial viability after cold plasma treatments is the disruption of the cell membrane through two events: (i) the oxidation of lipid components and (ii) the accumulation of charged particles on the cell membrane. The lipid oxidation at the first stage of reactive plasma species penetration generates malondialdehyde (MDA), which seriously affects intracellular components (proteins and DNA) via diffusion [27], initiating a cascade of damage in bacterial cells. Additionally, reactive and charged species bombarding the cell membrane break chemical bonds in compounds, especially hydrocarbons, forming holes and perforations in the cell membrane. This is also promoted by the electrostatic stress generated by accumulated charged species in the cell membrane, which finally results in an etching effect, disrupting the membrane [28].

Figure 3 shows the aforementioned formation of cavities and pores on the external structure (Figure 3B), ruptured cells at the midplane level of the divisome (Figure 3C), and cell collapse and flattening under the most intense treatment conditions (Figure 3C).

**Figure 4:**
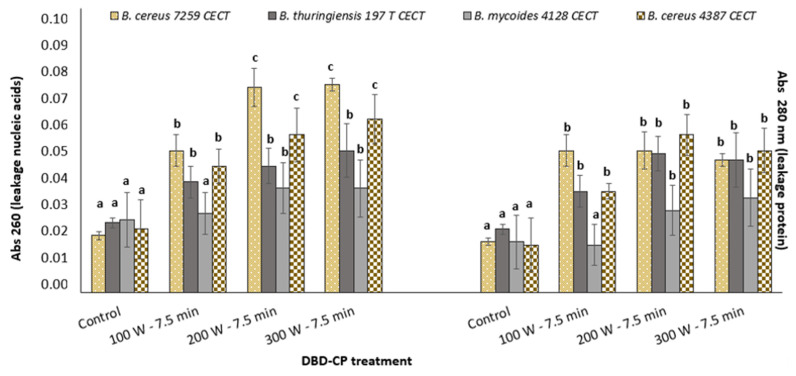
Spectrophotometric determination of nucleic acids and protein leakage caused by DBD-CP using O_2_ as a carrier gas on *B. cereus*, *B. thuringiensis*, and *B. mycoides* cells (control (untreated) and DBD-CP-treated cells). Superscript letters indicate significant differences (*p*-value < 0.05) in leakage values between species and groups (untreated versus treated cells).

Valuable studies have recently been published addressing the damage that cold plasma inflicts on bacterial cells, with images taken using SEM and confocal microscopy [15,16,17,18,29,30]. This kind of analysis (morphological and structural changes observed through images) can provide valuable information that can later be related to treatment parameters and give us an idea of how this cascade of events contributes to inactivation for each plasma–bacteria-specific binomial. Among the most recent studies, Charoux et al. [25] examined the damage/inactivation inflicted on *Bacillus subtillis* cells immersed in black pepper powder treated by atmospheric cold plasma jet technology (20 kHz; 15–30 kV, 3–20 min). Similar membrane rupture effects were detected by Charoux et al. [25] via SEM. This superficial damage was related to the subsequent leakage of intracellular components, which was observed through the progressive decay of the bacterial population during 48 h of storage [25]. In our study, possible intracellular component leakage was spectrophotometrically detected for the controls and DBD-CP-treated cells showing DNA and protein release in the media (Figure 4).

The results revealed significant differences between the effects of DBD-CP on *B. cereus* s.l. group species, with *B. cereus* strains specifically sensitive to plasma treatments, as demonstrated by increasing A260 and A280 values associated with plasma intensity increments. This indicates the continuous loss of cell membrane integrity caused by the DBD-CP active cloud effect. Lower A280 and A260 values were detected for *B. mycoides* cells exposed to DBD-CP, with treatments at 200 and 300 W required to observe any leakage events. The ability of cells to form bundles of chains, a unique set of spatial arrangements, in line with the higher resistance of *B. mycoides* to other stress factors, such as high salinity (NaCl < 7%), could validate our results regarding *B. mycoides* cell integrity maintenance under DBD-CP treatment. Previous studies also differentiate the effect of plasma on cells depending on the structure of the cell wall, considering Gram-positive bacteria more resistant than Gram-negative bacteria to the effect of reactive species integrating cold plasma active clouds [18,21,31].

### 3.2. Impact of DBD-CP on Integrity and Forming Capacity of Biofilm in B. cereus s.l. Group

**Figure 5:**
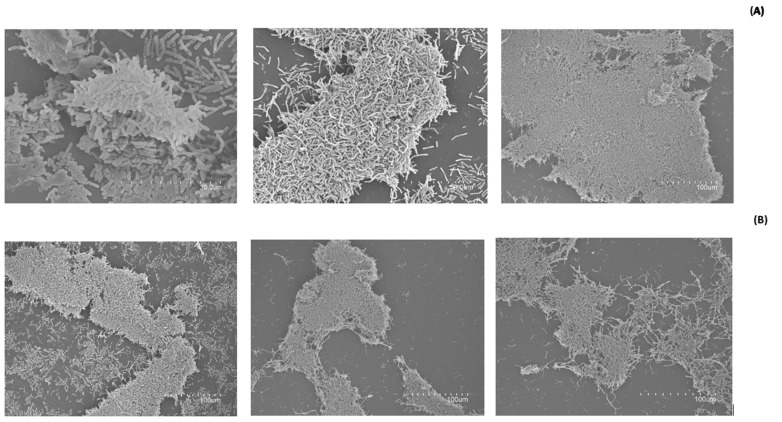
SEM images of biofilms formed by *Bacillus cereus* cells (4387 CECT). (**A**) Control biofilms (untreated). (**B**) Treated biofilms under different DBD-CP treatment conditions.

The biofilm-forming capacity is one of the main virulence factors associated with *B. cereus* pathogenic bacteria. Figure 5 shows the control biofilms formed by *B. cereus* 4387 CECT and DBD-CP-treated biofilms at 100, 200, and 300 W. The biofilm integrity assessment after DBD-CP application shows that the higher the plasma intensity, the higher the biofilm disintegration. There is a marked loss of continuity in biofilms treated with DBD-CP. The density of the biofilm matrix (integrated with bacteria and polymeric materials) decreases to a different degree depending on the treatment intensity.

To investigate how plasma affects the viability of cells immersed in biofilm, a cell viability assay based on reducing tetrazolium salt into formazan was used for the biofilms treated under the studied conditions and recovered in PBS. The results revealed an initial viability loss in the cells immersed in the biofilm of around 40% after treatment (100–300 W, 5 min) for all studied strains. However, after 8 h of incubation, this viability significantly recovered (biofilms subjected to 100–200 W for 5 min), comparable to what was achieved with the control biofilms. Nevertheless, with a more intense treatment (for 300 W), the loss of viability of cells immersed in biofilm did not translate into a progressive recovery in the detected metabolic activity. Inactivation and decay in cells immersed in DBD-CP-treated biofilms were confirmed according to the observed results at 300 W and 5 min. Therefore, in addition to the structural damage observed in the biofilm structure (network density and continuity), there was a significant percentage of damaged cells immersed in it that did not recover after 8 h, equivalent to the results obtained for planktonic cells (Section 3.1). In the same way, biofilms belonging to the *B. mycoides* strains were the most resistant to DBD-CP applications (Table 2).

**Figure 6:**
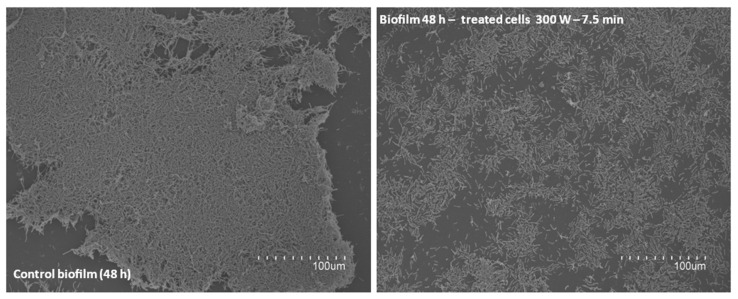
SEM images detailing biofilm-forming capacity of *B. cereus* (4387 CECT) after DBD-CP treatment.

To evaluate the biofilm-forming capacity of *Bacillus cereus* post-treatment, cells (viable + damaged) were recovered after different DBD-CP treatments. Figure 6 shows treatments at 300 W for 7.5 min indicated a lack of aggregation in the treated cells and a reduced capacity for adhering to the substrate (Figure 6).

Many studies have focused on evaluating the potential of cold plasma to inactivate and destroy biofilms. Most of these have been related to the destruction of biofilms caused by foodborne and clinical pathogens [32,33,34,35,36]. Lunder et al. [37] recently suggested that CP could be effective in reducing 72 h conformed biofilms but ineffective in reducing the integrity of 24–48 h formed biofilms caused by methicillin-resistant *Staphylococcus aureus* (MRSA). In addition, they found that the higher the treatment time (35 W–20 kV cold atmospheric plasma), the higher the cell viability loss inside the formed biofilm, independent of biofilm maturation (24, 48, and 72 h). *Listeria monocytogenes* and *Pseudomonas fluorescens* biofilms on vegetable surfaces completely disintegrated after just 5 min of atmospheric cold plasma (80 kV) [11]. Furthermore, Govaert et al. [34] achieved a significant level of reduction using an air–CP application (4 kV, 12 kHz) on biofilms formed by mixed species, in this case, by inactivating *L. monocytogenes* and *S. Typhimurium*, achieving values close to 1.5 and 2.5 log_10_ CFU/cm^2^ in biofilms.

The present results demonstrate that biofilm integrity is highly dependent on the treatment conditions applied (Figure 5), a relevant point related to the penetrability of plasma species in biofilm complex structures [33]. Considering the results regarding the biofilm-forming capacity of the Bacillus group after DBD-CP treatment, it can be inferred that aspects responsible for quorum sensing, such as the expression of genes involved in synthesizing autoinducing peptides, may be affected by plasma [38].

On the other hand, studies in relation to biofilm-forming capacity inhibition via CP are more extensive in the food industry [7,12,38]. Among the main studies focused on evaluating CP as a strategy to reduce the virulence of bacteria and assess the impact of treatment intensity on the biofilm-forming capacity of treated bacterial cells (viable + damaged) are Cui et al. [12], Ebrahimi-Shaghaghi et al. [38], and Guldimann et al. [39]. Ebrahimi-Shaghaghi et al. [38] described a reduction in *Candida albicans* biofilm-forming capacity after 10 W–120 s of cold atmospheric plasma application (ignited on helium + O_2_), within 40–60%, depending on the treatment applied (120–210 s). Cui et al. [12] confirmed a reduced biofilm formation capacity in *L. monocytogenes* after applying cold plasma ignited by pure nitrogen, reducing the metabolic activity of *L. monocytogenes* in biofilms by almost 55%. Guldimann et al. [39] demonstrated an increase in *sigB* expression (3.43 times) for *L. monocytogenes* after pure-nitrogen-ignited cold plasma (CNP), with *sigB* (encoded by sigB) essential factors for the initial adhesion in forming *L. monocytogenes* biofilm. Additionally, Guldimann et al. [39] found that CNP-treated bacteria reduced expression levels in *agr*A, *agr*C, and *agr*D by 73.3%, 67.9%, and 47.8%, respectively, compared with the values in untreated bacteria, which is closely related to interrupting the production of autoinducer peptides, the signaling molecules responsible the quorum sensing in biofilm formation.

## 4. Conclusions

DBD-CP applied at low pressure, using pure O_2_ as ignited gas, is an effective strategy for reducing bacterial species in the *B. cereus* s.l. group, with log_10_ cycles reduction levels (1–5 log_10_) that are higher than levels in *B. cereus* s.l. species doses in powder amylaceous contaminated foodstuffs (10–10^3^ CFU/g). Applying DBD-CP under the studied conditions was effective in reducing *B. cereus*, *B. thuringiensis*, and *B. mycoides* close to three log_10_ cycles under 100–300 W using short treatment times (1–5 min) at temperatures below 30 °C. The impacts of cold plasma applied under different studied conditions on the considered strains can be described as follows: (i) sublethal damage values between 40 and 90%, (ii) biofilm destruction, and (iii) biofilm-forming capacity reduction. Damage events (e.g., poration, elongation, cellular collapse, midplane disruption) were studied using SEM, revealing their influence on subsequent damaged population death. Damaged cells were found to evolve at 24 h after treatment, with DNA and proteins are released into the media, demonstrating decisive inactivation progression under the treatment. The planktonic cells and cells inside formed biofilms showed similar levels of viability reduction, demonstrating the effectiveness of DBD-CP’s ability to reduce the consistency of biofilm. The biofilm-forming capacity and biofilm structure were affected by DBD-CP in all studied strains. The present study is expected to provide valuable guidance for strategies to prevent and eradicate *B. cereus* s.l. group species in industrial food facilities, contributing to safety along the food chain and increasing the validation needed to finally implement CP technology in industrial facilities.

## Figures and Tables

**Table 1:** Sublethal damage (as a percentage) on *Bacillus cereus* sensu lato group cells caused by DBD-CP just after 100 W–5 min and 300 W–5 min treatments, and during a subsequent storage period of 24 h at 30 °C in BHIB.

DBD-CP Treatment Applied	Storage Time	*B. cereus* 4387 CECT	*B. cereus* 7259 CECT	*B. thuringiensis* 197 T CECT	*B. mycoides* 4128 CECT
100 W–5 min	0 h	98.1 ± 8.6 ^a^	78.2 ± 2.5 ^a^	45.6 ± 4.3 ^a^	74.4 ± 7.6 ^a^
	4 h	38.4 ± 2.6 ^b^	76.7 ± 3.1 ^a^	24.3 ± 2.8 ^b^	57.9 ± 3.4 ^b^
	8 h	35.3 ± 2.1 ^b^	43.1 ± 0.9 ^b^	26.1 ± 3.2 ^b^	1.73 ± 0.6 ^c^
	12 h	22.3 ± 1.4 ^c^	16.3 ± 4.2 ^c^	21.1 ± 2.1 ^c^	-
	24 h	26.7 ± 3.3 ^c^	24.3 ± 6.3 ^c^	13.1 ± 4.3 ^d^	-
300 W–5 min	0 h	78.2 ± 7.2 ^d^	81.5 ± 7.3 ^a^	42.1 ± 4.8 ^a^	92.5 ± 2.3 ^d^
	4 h	34.6 ± 11.8 ^b^	62.3 ± 3.5 ^d^	17.6 ± 3.2 ^d^	99.1 ± 6.8 ^d^
	8 h	39.4 ± 8.5 ^b^	7.4 ± 2.1 ^e^	21.3 ± 2.1 ^d^	92.4 ± 2.3 ^d^
	12 h	24.9 ± 1.5 ^c^	-	18.5 ± 3.6 ^d^	65.1 ± 7.4 ^a^
	24 h	23.8 ± 9.3 ^c^	-	16.2 ± 0.8 ^d^	31.7 ± 3.1 ^e^

^(a–e)^ Superscript letters indicate significant differences (*p*-value ≤ 0.05) between quantitative damage levels detected throughout the incubation period after DBD-CP application (for each strain and power level).

**Table 2:** Percentage of metabolic activity (%) detected using the XTT test on cells immersed in biofilms treated with DBD-CP (in relation to control biofilms).

*B. cereus* 4387 CECT	0	8 h	24 h
Control biofilms (untreated cells)	100	100	100
100 W–5 min	45 ± 2.3 ^a^	95 ± 5.3 ^c^	97 ± 3.7 ^c^
200 W–5 min	38 ± 3.5 ^a^	97 ± 2.4 ^c^	92 ± 1.2 ^c^
300 W–5 min	22 ± 1.8 ^b^	52 ± 3.3 ^d^	43 ± 4.8 ^d^
***B. mycoides* 4128 CECT**	**0**	**8 h**	**24 h**
Control biofilms (untreated cells)	100	100	100
100 W–5 min	65 ± 2.5 ^a^	87 ± 6.1 ^d^	98 ± 3.4 ^d^
200 W–5 min	58 ± 1.3 ^b^	91 ± 3.5 ^d^	95 ± 2.8 ^d^
300 W–5 min	36 ± 2.6 ^c^	45 ± 1.8 ^e^	52 ± 1.8 ^e^
***B. thuringiensis* 193T CECT**	**0**	**8 h**	**24 h**
Control biofilms (untreated cells)	100	100	100
100 W–5 min	43 ± 1.3 ^a^	84 ± 2.8 ^d^	90 ± 1.5 ^e^
200 W–5 min	32 ± 2.1 ^b^	81 ± 3.1 ^d^	95 ± 2.5 ^e^
300 W–5 min	22 ± 0.9 ^c^	27 ± 1.1 ^c^	38 ± 1.3 ^f^

^(a–f)^ Superscript letters indicate significant differences (*p* ≤ 0.05) between metabolic activity (rows) detected in treated biofilms under different DBD-CP conditions for each bacterial strain, analyzed with one-way ANOVA.

## Data Availability

The original contributions presented in the study are included in the article, further inquiries can be directed to the corresponding author.
